# The Alignment of Real-World Evidence and Digital Health: Realising the Opportunity

**DOI:** 10.1007/s43441-021-00288-7

**Published:** 2021-04-29

**Authors:** Sajan Khosla, Maurille Feudjo Tepie, Mark J. Nagy, George Kafatos, Michael Seewald, Stephanie Marchese, Johan Liwing

**Affiliations:** 1 Real-World Evidence Center of Excellence, AstraZeneca, Cambridge, UK; 2 Center for Observational Research, Amgen, Cambridge, UK; 3 Eli Lilly and Company, Lilly Corporate Center, Indianapolis, IN USA; 4 IQVIA, London, UK; 5 XNK Therapeutics, Stockholm, Sweden

**Keywords:** Digital health, Real-World Evidence, Real-Word Data, Health data, Guidelines, Wearables

## Abstract

In the new era of healthcare digitalization, there is a golden opportunity in the overlap between digital health and Real-World Evidence (RWE). In this commentary, we define RWE and digital health and investigate their intersection. We describe the stages in the RWE value chain critical to the evidence generation process, how these stages change with new digital technologies and the opportunities and challenges that arise from how these stages evolve—including their application for stakeholders such as patients, physicians and regulators. We also discuss the current published guidelines and frameworks regarding digital health. We categorise these publications in terms of their clarity as “Extensive”, “Intermediate” or “Basic” and according to whether they encompass all levels of digital health or are more focussed in their guidance. Finally, we provide recommendations to increase synergy between RWE and digital health.

## Introduction

In recent years, technological capabilities have expanded and person-generated digital health data use has increased in importance and this could now complement traditional electronic health data. Furthermore, the recent trend for value-based, patient-centric care provides an opportunity for person-generated digital health data to play a fundamental role in healthcare systems, and in the generation of Real-World Evidence (RWE). As such, the concepts of digital health and RWE are intrinsically linked, with digital health being able to provide RWE and RWE requirements and gaps creating a need for data generation by new and innovative means.

Many definitions of RWE and digital health exist, leading to potential misalignment over the terminology. Alignment is, therefore, needed to ensure that stakeholders are using a common nomenclature. We will now consider definitions of RWE and digital health before providing commentary on the intersection between the two.

We define RWE as “Insights generated from Real-World Data using appropriate scientific analytics with the intention to support a claim or belief, for which a hypothesis is usually formulated in advance”. Existing use cases for RWE include demonstrating safety and value, pricing and market access and improving the clinical development process [1]. Real-World Data (RWD), from which RWE is derived, is defined as follows: “Longitudinal patient-level data captured in the routine management of patients (for care, cost management or public health) which can be repurposed to study the impact of healthcare interventions”.

**Figure 1.**
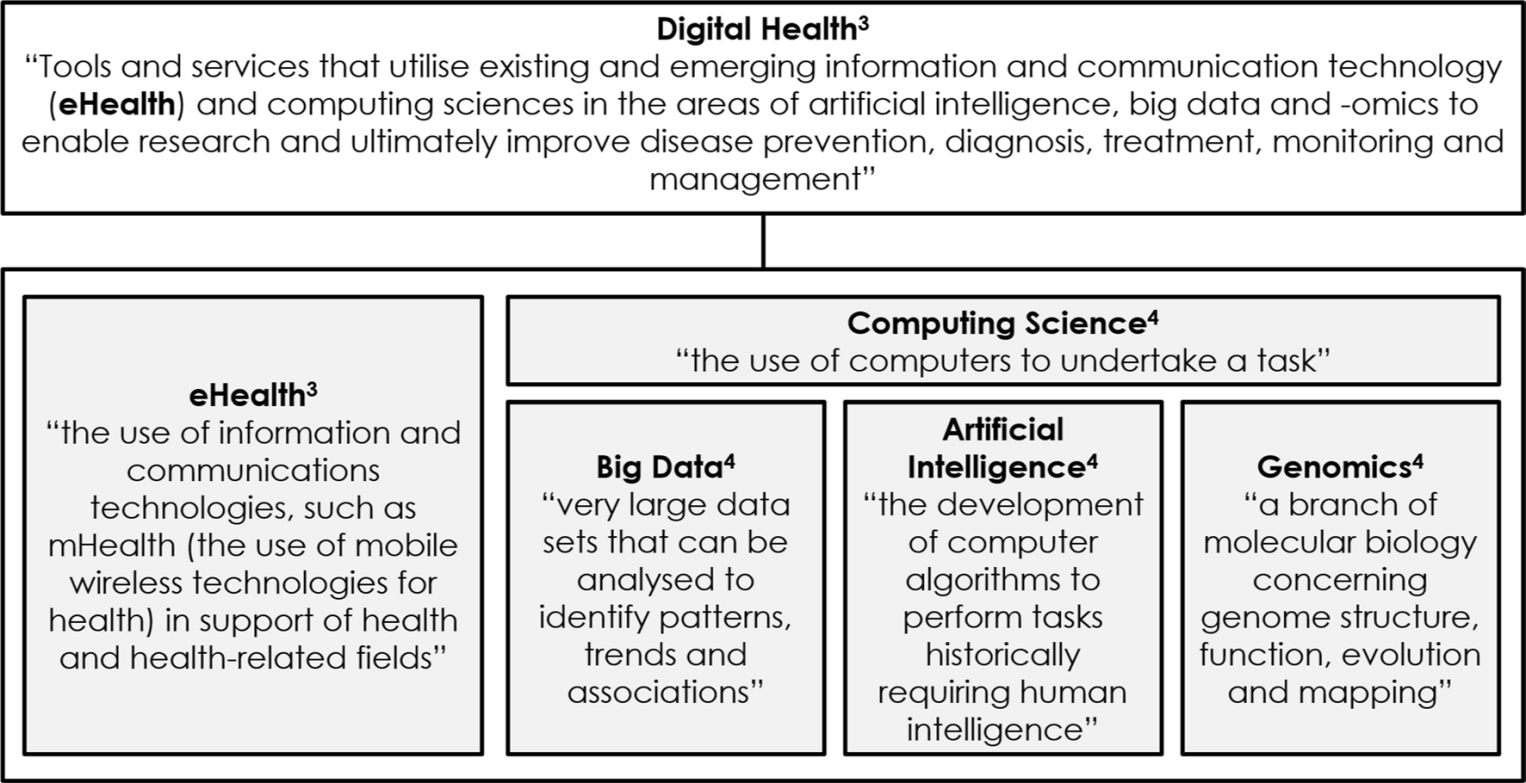
Hierarchy definition of Digital Health. The constituent parts of Digital Health are the use of information and communication technology (eHealth) and of computing sciences in the areas of Artificial Intelligence, Big Data and genomics in support of health [3, 4].

With advances in digital health, namely, tools to generate data, decision support tools or medical products to support health, comes the ability to generate novel RWE. Digital health also has multiple definitions ranging from the European Commission’s definition “Digital Health and care refers to tools and services that use information and communication technologies (ICTs) to improve prevention, diagnosis, treatment, monitoring and management of health and lifestyle” [2] to the WHO definition “An overarching term that comprises eHealth (which includes mobile Health), and emerging areas, such as the use of computing sciences in the fields of artificial intelligence, big data and genomics” [3]. Combined, the European Commission and WHO definitions provide a concise definition, but do not explicitly incorporate the role of research. We propose a hierarchy approach, as illustrated in Fig. 1, to describe “Digital Health”. First, we define digital health as “Tools and services that utilise existing and emerging information and communication technology (eHealth) and computing sciences in the areas of artificial intelligence, big data and genomics to enable research and ultimately improve disease prevention, diagnosis, treatment, monitoring and management,” to which electronic information, communications (eHealth) and computing science (big data, artificial intelligence and genomics) contribute. Finally, we define digital health data, a subset of digital health encompassed by all contributing components, as “The (passive or active) collection of health data via a digital tool, device, wearable or app”. Digital health data can be transformed into “digital health evidence by utilising computing sciences and other appropriate analytical methods”.

Considering the increasing role of digital health in evidence generation, international health bodies have efforts underway to align on the role of digital health. For example, the International Coalition of Medicines Regulatory Authorities (ICMRA) “Innovation Project” has delivered insights on how AI, big data and genomics impact on healthcare [5]. More recently, ICMRA activities have focussed on the use of RWE in relation to COVID-19 to understand treatment effectives and vaccine monitoring. Ongoing efforts include establishing data source networks with common data models to create patient cohorts for analysis and the implementation of safety and monitoring systems.

## Changes, Opportunities and Challenges in the Era of Digital Health

**Figure 2.**
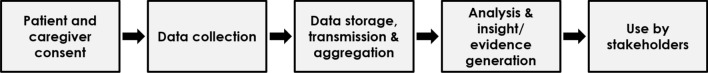
Stages in the RWE value chain. Areas that are critical in the evidence generation process are patient and caregiver consent, data collection, data storage transmission & aggregation, analysis and insight/evidence generation and use by stakeholders.

Looking at the intersection of digital health and RWE, there are critical areas in the evidence generation process that we define as stages in the RWE value chain, (Fig. 2). Here, we start with the patient as our source of information, from whom we collect data. That data are then stored and transmitted so it can be aggregated with data from other patients. Finally, data are analysed to generate RWE for final use by stakeholders. These use cases could include, for example, the creation of external comparator arms for clinical trials or to support post-authorisation safety commitments.

We have further explored how digital health is changing these stages and the opportunities and challenges that arise (Table 1). We anticipate changes will impact all stages of RWE generation, resulting in many new opportunities. The proliferation of digital devices will drive changes in the first three stages as patients engage with health apps and digital devices, such as wearables, to generate data for analysis. “Analysis & insight/evidence generation” and “Use by Stakeholder” will see the greatest change due to the volume and novelty of analysis that comes with having not only more real-time data and novel data variables, but also new analytical techniques, such as AI and machine learning. The potential increase in generated evidence will be profound with greater ability to identify risk factors within specific patient populations and previously unreported adverse events, and, better understand disease incidence across geographies, through the use of complex algorithms in new large datasets. This will feed into how stakeholders use evidence to support submissions, make regulatory decisions, and will, ultimately, produce better outcomes for patients.

**Table 1:** How RWE Will Change with the Introduction of New Digital Technologies and Opportunities and Challenges Arising from These New Advancements.

Stage	How stages will change		Opportunities and challenges	
	unchanged	Changes	Opportunities	Challenges
Patient and caregiver consent	Legal requirements for *confidentiality recruitment* into studies Need for *consent* according to the Helsinki declaration	New ways for patient *consent* (e.g. electronically) *Patient behaviour* as patients become more active in data generation Increased opportunities for *patient engagement* Increased *holistic disease/treatment view*	New insights to support *informed HCP/patient conversation* *Patient centricity* – putting the patient at the centre of data generation activities and insight application Digital PROs and COAs (Clinical Outcome Assessment) will be able to *supplement and improve* health data, thus improving *completeness and quality* Patients will be connected to the *wider patient community and healthcare stakeholders* with further opportunities for participation via *social media* Increased ability to reach out to patients for *involvement in medical research*	Patient comfort with *data and information sharing* Patient concerns over *data misuse* and *security* *Patient selection “bias”* – potential to under-represent less digitally literate patients [7] Physicians *overload with the availability of new data* *Media and social media misinformation* (e.g. anti-vaccination) Multiple interfaces and solutions are *not linked*
Data collection	Respect for *GDPR, and other data privacy legislation *[6] Data collection from *traditional data sources* e.g. physician reports, health records, claims data etc	New ways to collect data and *new data types/sources* *Technical infrastructure* requirements *Location* of data collection can be decentralised and closer to the patient Ability to collect data *24/7*	*More direct relationship* between patient and technology developer and other healthcare stakeholders *New types of data* direct from patients including PROs/COAs/adherence/ genetics (e.g. *23 and me*) Potential reduction of the *costs* associated with traditional data collection through automation *Real-time* data updates	Implementation of necessary *technical infrastructure*, such as capacity to deal with high data volumes Patient-generated data not validated by physicians *may be of lower usability* *Maintaining patient engagement* in digital activities *Patients’ willingness* for all stakeholders to have *access* to all information *De-identification/pseudonymisation* of data compliant with legislation
Data storage, transmission & aggregation	*Legal framework* surrounding data processing	*Server capacities* to store high volume/velocity/variety data (cloud storage) New ways to *process* data Potential to *link data* from digital devices/novel sources to health records	*Linking data* from variable sources for increased insights More *comprehensive data* representing patient health and wellness	*High volume of data* to be stored/ transmitted may be too much for existing infrastructure *Interoperability* across multiple systems and technologies Lack of a *unique patient ID* needs to be overcome to allow data linkage – especially with de-identified/ pseudonymized data Transmission requires reliable *internet connection* Meeting necessary requirements *for data security, integrity and harmonisation,* including concerns over monetization of patient data *Governance rules* not allowing data linkage
Analysis & insight/ evidence generation	Need for analytics to transform data into robust (digital) *evidence*	Use of specialised *analytical tools* that can handle large volumes of data Use of *analytical techniques* that can fully utilise these new data e.g. artificial intelligence (AI) tools *New types of insights* possible *Validation of new data sources and new digital endpoints* used in analysis	Greater potential for *artificial intelligence/machine learning* with larger data sets better suited for these techniques Availability of *new patient-relevant endpoints,* giving a more holistic view of the patient Greater insight robustness from *higher volume of data (reduced uncertainty)*	Shortage of *technical/analytic skills* in industry Lack of and *stakeholder experience* in digitally derived elements *“Black box”* problem of highly complex new analytical techniques Uncertainty over *validation/confidence* in the new analytical methods from stakeholders *Maintaining patient anonymity* in free text
Use by stakeholders	Overarching *payer/regulator needs* (safety, efficacy, etc.) Requirement for *post-marketing commitments and pharmacovigilance*	Wider variety of *data sources* to use Additional data will increase *clinical decision support* *Approval process* for digitally derived data *Patient treatment process* through device usage and clinical decision support Transitioning from *population health management* to *patient-level support*	Increased potential to generate *new types of evidence* with payors/ regulators (e.g. virtual trials) *Reimbursement of apps* by payors [6] Closer to *real-time evidence and insight generation* Improved *patient care* through *clinical decision support* and *responsive pathways* Increased *shared decision-making* between patient and physician Greater level of *personalised medicine* in relation to treatment strategy Further support and application of *health screening* and *preventative health* *Improved trial recruitment* through better, more *granular patient-level data* *Clinical trial design* may move towards more virtual trials Opportunity to include *data-driven feedback loops* *“Beyond the pill”* services (e.g. “Sleepio” app) [8]	Uncertainty over stakeholder *acceptability* of digitally derived insights *Stakeholder’s ability* to transfer, receive, store and process large volumes of data *Inadequate application* of digital health could drive up healthcare costs

The increasing contribution of digital health will not be without challenges. Barriers in relation to patient comfort with data sharing and data security will need to be addressed, stakeholder acceptance of digitally derived data will need to be overcome, and the infrastructure to accommodate large datasets will need to be implemented. There will also need to be alignment across stakeholders on the requirements of digital health and RWE. As such, regulators are now publishing guidelines for RWE and digital health data.

## Current Key Guidelines and Recommendations

In a rapidly changing environment, it is important to understand the regulatory and RWE requirements for digital health. In recent years, regulators and international healthcare bodies have published guidelines for digital health. Their publication often follows digital health strategies from national governmental agencies, such as Ministries of Health. These are aimed at all stakeholders and outline a country’s vision for digital health and therefore tend to be broad, aspirational and limited to key priority areas such as telehealth, infrastructure and EHRs.

A review of some recent key digital health guidelines, covering the EU and USA (Table 2), found that these fall under two categories: high-level overviews that encompass all aspects of digital health; or, detailed guidance for specific constituent components of digital health (e.g. health apps). Key guidelines falling under the “high-level” category include those from: NICE (UK) [9]; FDA (USA) [10]; European Commission (EU) [11]; EMA (EU) [12]; and, WHO (global) [3, 13]. While these are strategic in nature, they also include recommendations on how to efficiently utilise digital technologies in healthcare. For example, The EMA’s Strategic Reflection [12] includes recommendations on where digital advances can be leveraged, such as the facilitation of novel manufacturing process. The same plan also provides recommendation on how to advance digital technologies, such as the establishment of dedicated AI laboratories in which to test the application of digital technologies. Furthermore, the plan details recommendations for accommodating the needs of decision makers, for example, incorporation of payer needs into development plans and discussions with HTAs in the production of guidance for evidence generation review. Other guidelines provide more detailed guidance on specific aspects of digital health. Many of these focus on topics, such as health apps (e.g. HAS, France [14]; MHRA, UK [15]), telemedicine (e.g. Italian Ministry of Health [16]) or AI (e.g. FDA, USA [17]). The MHRA’s guidance on medical device software and apps [15] provides guidance as to when an App would be considered a medical device in order to allow developers to understand requirements surrounding CE marking in addition to advice on how to address the CE marking process, including post-market surveillance. Important to note here is that these guidelines do not cover the data itself (i.e. raw data, data collection) and references to other legal requirements concerning data protection are provided.

**Table 2:**
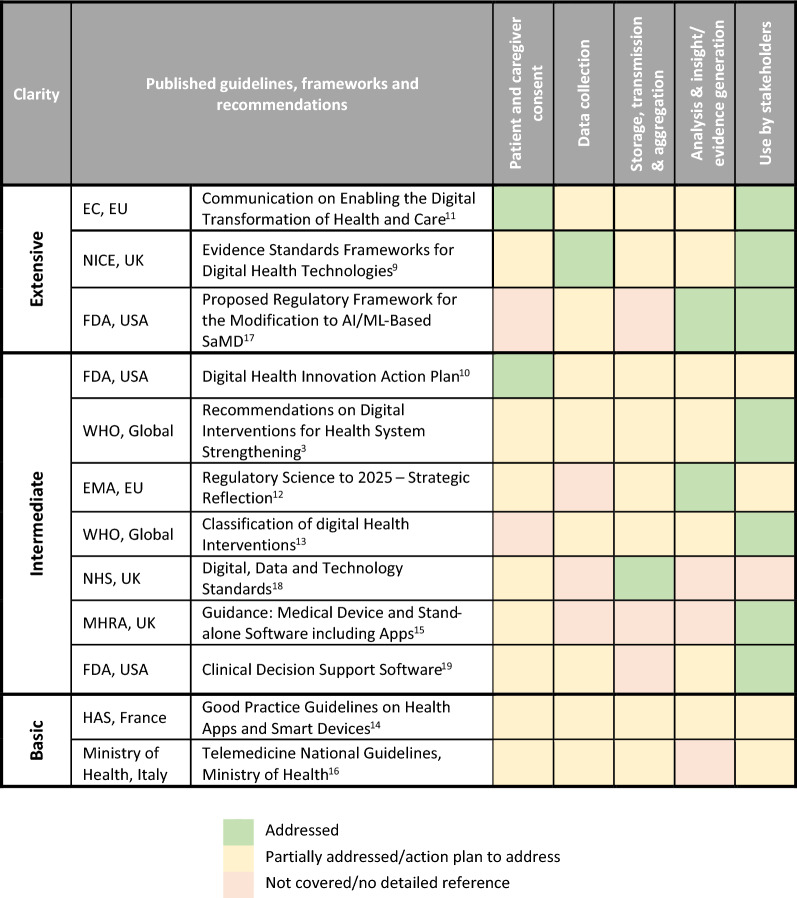
Stages of RWE Value Chain Mapping to Selected Key Digital Guidelines, Frameworks and Recommendations.

If we consider the RWE value chain stages (Fig. 2) and their mapping to the guidelines (Table 2), the overlap between digital and RWE generation becomes clearer, with many guidelines attempting to provide at least some clarification across the stages of the value chain, with a particular focus on end case uses for digital health. Where no clarity is provided on specific stages, it is often because the guidelines were focussed on a specific aspect of digital health where other stages were less relevant. For example, data collection and data storage, transmission and aggregation would fall under data protection legislation and are not the responsibility of the MHRA so are not included within the MHRA guidance on medical device software and apps [15].

RWE is not explicitly referred to in the majority guidelines although some bodies have provided some clarity. The NICE Evidence Framework for Digital Health Technologies [9] fully describes the evidence standard frameworks which need to be developed for effectiveness and economic impact, two key requirements for payers. Furthermore, The FDA proposed framework [17] outlines evidence requirements relating to clinical association, analytical validation and clinical validation. Although not providing evidence requirements for digital health, the EMA’s Strategic Reflection [12] also outlines a strategic goal to drive collaborative evidence generation and the need for a regulatory framework for clinical data generation from digital technologies.

## Recommendations for Increasing Synergy Between RWE and Digital Health

Digital health and RWE are rapidly evolving areas in healthcare in a reciprocal way, with digital data generating RWE and RWE driving further digital innovation and acceptance. However, both have challenges (Table 1), for example, patient comfort with data sharing, regulator/payer acceptability of digitally derived endpoints and lack of clarity from regulators regarding RWE requirements from digital health. In some instances, stakeholders are already working towards addressing these issues. For example, some regulatory guidelines are open for feedback from other healthcare stakeholders. These guidelines are being updated as understanding and technology evolve and as the requirement for further clarity grows. However, the extent to which individual guidelines provide the necessary detail on generating RWE and how it will be assessed from digitally derived data is limited.

Regulators are a significant stakeholder in digital data and some are being proactive in the areas of digital health through the publication of guidelines. However, further guidance and transparency over historic regulatory decisions is required from digitally aware entities and regulators less engaged with digital data need to start formulating in-depth guidelines on specific topics with input from industry and academia. This will enable drug companies and medical device developers to ensure the appropriate variables are used to conduct analysis and that AI/ML techniques applied during analysis are robust. Furthermore, setting specific standard requirements and frameworks for reviewing digitally derived RWE would ensure consistency across individual reviewers. Finally, regulators could themselves benefit from technological advances in digital health. As outlined in the EMA’s strategic reflection [12], scientific evaluations could be improved through the utilisation of AI in the decision-making process.

Clarity also needs to be provided by payers. Although not discussed in this paper, payers will need to provide guidance on the requirements for reimbursement of digital technologies, for example, prescription of healthcare-related apps which would otherwise incur a direct cost to the patient or be unavailable. This guidance will not only need to be provided to manufacturers and developers, but also to clinicians and institutions so they are aware which digital products are eligible for reimbursement. As with regulators, payers could also benefit from alignment on how digitally related RWE is accessed as this would increase internal understanding and efficiencies post-marketing commitments for digital technologies relating to effectiveness could influence future payer decisions.

Provision of guidelines to healthcare professionals and their institutions will educate them in the evidence requirements of digital health products, such as apps and wearable devices, and clinical decision support tools, and thus gain their acceptance so that they might prescribe apps to their patients or use the outputs from clinical decision support tools to influence diagnosis and treatment. Furthermore, access to a library of apps which can be prescribed, like the NHS Apps Library in the UK [20], would facilitate ascertaining which healthcare apps could be beneficial for patients. For example, the Sleepio App uses cognitive behavioural therapy for insomnia to help patients sleep better without the need for sleeping pills [21].

Finally, healthcare systems need to become integrated to allow the sharing of both traditional and novel data across multiple care settings to feed into clinical decision support tools. For example, data systems incorporating data from primary, secondary and tertiary care with the flexibility to incorporate data feeds from health apps and wearables will enable clinical support tools to more accurately facilitate clinician treatment decisions. Similarly, health devices using AI will need to connect with health data systems to ensure that the data underlying the output of the product is correct, up to date and contains all variables or proxies required to undertake the required analysis.

Key to the validation of novel endpoints and analytical processes, such as AI and machine learning, will be engagement with academia. Academics will enable novel endpoints to be defined and provide evidence supporting the use of newly emerging analytical methodologies and as such will be in a position to independently validate the RWE generated through digital means across the relevant stages of the RWE value chain including the selection of clinically relevant data variable collection, appropriate use of robust analytical processes and correct insight generation. Similarly, academics will be able to validate the outputs of clinical decision support tools through protocolised research studies investigating how outcomes vary between patients treated according to these tools, versus clinician choice. As a consequence of academic validation, acceptance will impact across stakeholders, such as regulators and research-engaged healthcare professionals.

The pharmaceutical industry will also play a valuable role in ensuring RWE and digital health synergy. It will be their responsibility to fully identify the gaps in the data generated by digital technologies and understand how these can be addressed in order to generate relevant, high-quality RWE. Furthermore, it will require pharmaceutical companies to support regulators and payers in the creation of guidelines to ensure that the requirements for digitally generated RWE are feasible and aligned across regulators and payers. Thus, it will be imperative for the formation of collaborative partnerships between industry, technology companies and healthcare bodies to generate the data required and deliver innovative and effective solutions. Furthermore, clinical trials, in partnership with academic institutions and healthcare providers, will be needed to demonstrate the benefits of digital health and post-launch evidence of these benefits will need to be continuously updated as further data are generated in the real-world setting.

## Conclusions

In this commentary, we have provided clarity over the definitions of RWE and digital health and discussed their intersection and challenges and opportunities that will arise as digital health advances across the RWE value chain. We have provided high-level analysis on the guidelines surrounding digital health and have provided recommendations on what needs to be done to ensure success. While we have focussed on the EU and the USA, it is worth noting that other markets, such as China, are widely expanding their healthcare digital capabilities and in the coming years will have an influential role to play in the utilisation of digitally generated health data.

In conclusion, the opportunities of digital health will benefit all stakeholders through the provision of a holistic view of disease and treatments and a means by which new data and insights can be generated. However, many challenges need to be addressed in order to realise the benefits of the RWE and digital health relationship.
